# Vocal Acoustic Features as Potential Biomarkers for Identifying/Diagnosing Depression: A Cross-Sectional Study

**DOI:** 10.3389/fpsyt.2022.815678

**Published:** 2022-04-28

**Authors:** Qing Zhao, Hong-Zhen Fan, Yan-Li Li, Lei Liu, Ya-Xue Wu, Yan-Li Zhao, Zhan-Xiao Tian, Zhi-Ren Wang, Yun-Long Tan, Shu-Ping Tan

**Affiliations:** Peking University HuiLongGuan Clinical Medical School, Beijing Huilongguan Hospital, Beijing, China

**Keywords:** depression, acoustic characteristics, MFCC, biomarker, zero-crossing rate

## Abstract

**Background:**

At present, there is no established biomarker for the diagnosis of depression. Meanwhile, studies show that acoustic features convey emotional information. Therefore, this study explored differences in acoustic characteristics between depressed patients and healthy individuals to investigate whether these characteristics can identify depression.

**Methods:**

Participants included 71 patients diagnosed with depression from a regional hospital in Beijing, China, and 62 normal controls from within the greater community. We assessed the clinical symptoms of depression of all participants using the Hamilton Depression Scale (HAMD), Hamilton Anxiety Scale (HAMA), and Patient Health Questionnaire (PHQ-9), and recorded the voice of each participant as they read positive, neutral, and negative texts. OpenSMILE was used to analyze their voice acoustics and extract acoustic characteristics from the recordings.

**Results:**

There were significant differences between the depression and control groups in all acoustic characteristics (*p* < 0.05). Several mel-frequency cepstral coefficients (MFCCs), including MFCC2, MFCC3, MFCC8, and MFCC9, differed significantly between different emotion tasks; MFCC4 and MFCC7 correlated positively with PHQ-9 scores, and correlations were stable in all emotion tasks. The zero-crossing rate in positive emotion correlated positively with HAMA total score and HAMA somatic anxiety score (*r* = 0.31, *r* = 0.34, respectively), and MFCC9 of neutral emotion correlated negatively with HAMD anxiety/somatization scores (*r* = −0.34). Linear regression showed that the MFCC7-negative was predictive on the PHQ-9 score (β = 0.90, *p* = 0.01) and MFCC9-neutral was predictive on HAMD anxiety/somatization score (β = −0.45, *p* = 0.049). Logistic regression showed a superior discriminant effect, with a discrimination accuracy of 89.66%.

**Conclusion:**

The acoustic expression of emotion among patients with depression differs from that of normal controls. Some acoustic characteristics are related to the severity of depressive symptoms and may be objective biomarkers of depression. A systematic method of assessing vocal acoustic characteristics could provide an accurate and discreet means of screening for depression; this method may be used instead of—or in conjunction with—traditional screening methods, as it is not subject to the limitations associated with self-reported assessments wherein subjects may be inclined to provide socially acceptable responses rather than being truthful.

## Introduction

Depression is a serious mental illness that can result in a significant decline in a patient’s social functioning and quality of life. According to a 2019 epidemiological survey in China, the lifetime prevalence of depression was 3.4%, and the 12-month prevalence was 2.1%—both of which are higher than the prevalence rates for most other psychiatric disorders (1). Currently, the diagnosis of depression is primarily dependent upon the subjective judgment of experienced psychiatrists. However, accurately distinguishing negative emotions from depression is challenging, and objective and effective auxiliary diagnostic methods for depression are lacking. These are among the possible reasons that depression is easily missed. For example, in primary care settings, only one-third of people with mild depression are correctly diagnosed (2). After experiencing a first depressive episode, the recurrence rate within 3 years is more than 30%, and some patients have recurrent episodes; thus, the course of the disease can be chronic, placing a heavy burden on both patients and society (3, 4). Consequently, the use of any potential objective biomarker for accurately diagnosing depression warrants in-depth investigation.

The speech of depressed patients is different from that of normal people. The patients usually show lower intonation and less speech. Many studies have suggested that there are abnormal acoustic features in depressed patients (5, 6). This suggests that acoustic features may be an objective biomarker of depression. Therefore, to determine an appropriate biomarker to investigate, we considered the question, “What is the physical process of speech?” The brain organizes prosodic information and produces neuromuscular instructions that control the activities of muscles and tissues related to phonation movement. Next, the airflow stream out of the lungs either causes the vocal cords to vibrate (when the glottis is closed) or passes through the vocal cord smoothly (when the glottis is open). The oropharyngeal muscle forms the main channel of phonation, which is equivalent to a filter that can amplify or attenuate the sound of a specific frequency (7).

Regarding the content of speech, it mainly consists of two different types of information: “what” is being said, including the literal meaning of the information expressed, and “how” it is said, which connotes the emotion and rhythm information in the expression. The speech of a depressed patient can convey not only verbal information, such as negative, pessimistic, and suicidal ideas, but also considerable non-verbal information, including slow reaction and speech, lack of attention, and low volume of voice. “Voice,” in this context, is a non-verbal form of emotional expression (8). Emotion affects speech by changing the physiological characteristics of the vocal system (9), breathing, and muscle tone while listeners perceive the emotion of the speaker through specific neural pathways (10).

Depressed patients tend to show less speech production, have a low intonation, and pause often, all of which are characteristics of this mental disorder. Acoustic characteristics of speech that people can hear directly, such as the length of a pause, pause variability, and the speech/pause ratio, differ between people with and without depression, and studies have verified that some of these characteristics indicate depression severity (11–14). These reported findings suggest that voice analysis may be used to gauge the severity of depression objectively. However, the aforementioned characteristics are easily affected by unstable environmental factors. Therefore, the mel-frequency cepstral coefficient (MFCC) is often considered a superior method for distinguishing differences in voice emotion characteristics and analyzing the subtle differences in voice emotions perceived by human ears (15).

MFCCs can be used to identify changes in the vocal tract (16) and can be calculated in the following steps: (1) calculating the fast Fourier transform spectrum from the frequency, (2) extracting filter bank output of mel scale allocation according to human auditory features, and (3) obtaining the cepstrum coefficient from the discrete cosine transformation. Ozdas et al. (17) found that MFCC has an accuracy of 75% in distinguishing between depressed patients and normal controls. Akkaralaertsest and Yingthawornsuk (18) also believed that there were characteristic differences in MFCC of depressed patients’ speech that could help identify depressed patients with or without high suicide risk, to some extent.

In addition to the MFCC, other characteristics can indicate the voice mood. The fundamental frequency (F0) is the basic frequency generated by the vibration of the vocal cords. Results from a 4-week sertraline/placebo double-blind controlled trial showed that the mean fundamental frequency of the depression group that responded to the treatment increased from baseline; meanwhile, the frequency remained consistent or decreased from baseline for the depression group that did not respond to the treatment (19). Additionally, the zero-crossing rate (ZCR), which refers to the number of times a signal passes through the zero axis in a certain time, can be used to identify voiceless and voiced sounds that are characteristic expressions of phonetic emotions. A Chinese study found the ZCR has an accuracy of 67–70% to distinguish depressed individuals from those without depression (20). However, a Japanese study found no significant difference in ZCR between the depression and normal groups (5). Furthermore, although the harmonic-to-noise ratio (HNR) reflects the ratio of the sound signal strength to noise signal strength, few studies have investigated the relationship between depressive symptoms and acoustic characteristics, such as ZCR, MFCC, HNR, and F0.

Additionally, although previous studies that used small sample sizes and included limited acoustic characteristics have shown that depressed patients present abnormal acoustic characteristics, no studies to date have verified the correlation between acoustic characteristics and clinical symptoms. Hence, there is a lack of explanation for the psychopathology of abnormal acoustic characteristics. It is unclear whether acoustic features can partly reflect the severity of emotional symptoms and whether the severity of depression can be evaluated by certain acoustic features.

To address this limitation, the present study surveyed a relatively large sample of depressed patients and healthy controls. Using MFCC, ZCR, HNR, and F0, which are known to reflect the emotional changes of the voice, we aimed to verify the relationship between acoustic characteristics and severity of depression and the significance of acoustic characteristics in disease discrimination. We also conducted a preliminary exploration of the psychopathological mechanism of abnormal acoustic characteristics.

We hypothesized that the acoustic characteristics of depressed patients and normal people differ and are related to depressive symptoms and that acoustic characteristics can help distinguish depressed patients from normal individuals to some extent. If acoustic characteristics can be used as objective biomarkers of depression to provide a diagnostic reference, it may help decrease the rate of misdiagnosis and missed diagnosis, promote early identification and treatment of depression, and ultimately improve the patient’s prognosis. Since acoustic characteristics can be obtained automatically, exploring the acoustic characteristics that can predict the severity of anxiety and depression was conducive to promoting the intelligent evaluation of depressive disorder severity. Moreover, it can also promote the development of artificial intelligence ability to identify depression and provide Internet-based medical treatment to patients, which can have far-reaching implications.

## Materials and Methods

### Participants

We recruited 71 patients from Beijing Huilongguan Hospital from 2018 to 2020. An experienced psychiatrist conducted detailed mental and physical examinations of the participants. The inclusion criteria were as follows: (1) diagnosed with depression according to the Diagnostic and Statistical Manual of Mental Disorders (DSM–IV), (2) aged 18–60 years, (3) attained junior high school education or above, and (4) able to read Chinese fluently. The exclusion criteria were as follows: (1) diagnosed with severe cerebrovascular disease, brain injury, and other nervous system diseases; (2) having a serious physical disease; (3) having accompanying substance abuse (except tobacco) problems; and (4) diagnosed with acute nasolaryngology and respiratory system diseases affecting the vocal cords. Sixty-two healthy controls with no current or previous mental illness were recruited from the surrounding community and screened by the Mini-International Neuropsychiatric Interview. The remaining inclusion and exclusion criteria were the same as those used in the patient group.

This study was approved by the Ethics Committee of Beijing Huilongguan Hospital. A specific psychiatrist explained the details of the study to all the participants; meanwhile, their information was strictly confidential. Audio data would be stored on a specific hard disk. Participants took part in voluntarily and there would be no penalty for withdrawal at any time. Written informed consent was obtained from all participants.

### Measures

All participants provided their general information, and the depression group completed clinical symptom evaluation questionnaires, including the 17-item Hamilton Depression Scale (HAMD-17), Hamilton Anxiety Scale (HAMA), and Patient Health Questionnaire (PHQ-9). The HAMD-17 is a commonly used depression rating scale comprising 17 symptom items across five domains including anxiety/somatization, weight, cognitive impairment, retardation, sleep disturbance (21, 22). The HAMA is a commonly used 14-item anxiety rating scale for measuring “physical anxiety” and “mental anxiety;” each item is scored from 0 (not present) to 4 (very severe) (23). We also used the PHQ-9, a simple and effective 9-item self-assessment tool for depression wherein each item is rated on a scale from 0 to 3 (24). Higher scores on these scales indicate greater severity of symptoms.

All participants were seated in a noise-controlled room (background sound less than 30 dB). After a 3-min rest period, voices were recorded using a fixed ISK BM-5000 microphone (sampling frequency: 44 kHz, bit rate: 1,058 kbps). The participants were asked to read three paragraphs in their natural tone. Each paragraph contained about 200 Chinese characters, which described one of the three different emotions: positive, negative, and neutral. The positive emotion text depicted scenes from the Chinese New Year and included words such as “happy,” “smile,” “play games,” “delicious,” “singing and dancing,” and so on. The negative emotion text included “punishment,” “trouble,” “worry,” “anxious,” “worry,” “slander,” “pain,” “dark clouds,” and so on. The neutral emotion text described a bridge using location, shape, and other descriptors that did not contain any emotion-related words. Word stimulations can induce different emotional and physiological responses (25, 26) and might influence acoustic differences between the depression group and control group (6). However, it is not clear whether the different emotional stimuli could play a role in the discriminant analysis, so we explored this further. After reading a paragraph, the participants were asked to rest for 1 min before moving on to the next paragraph. Voices were analyzed with OpenSMILE v2.1.0 (27) using the feature set of INTERSPEECH 2009 Emotion Challenge pre-set configuration (IS09_emotion.conf). The mean values of the following acoustic features within the utterance segments were calculated for the 12 dimensions of MFCC, ZCR, HNR, and F0.

### Statistical Analyses

All statistical analyses were performed using the SPSS 21.0. For normally distributed continuous variables, the independent sample *t*-test was used for comparison between groups. Categorical data were analyzed using the chi-square test. Multivariate analysis of variance was used to compare the differences in acoustic characteristics between groups, with gender, age, and education level as covariates. To address the problem of multiple comparisons (15 acoustic features), a false discovery rate (FDR) procedure was further performed at a *q*-value of 0.05 over all acoustic features together. Spearman correlation analysis was performed to explore the relationship between depression symptom scores and acoustic characteristics. Further, multiple linear regression was conducted to explore the relation between clinical symptom and acoustic characteristics.

To choose the more suitable discriminant model, the discriminant effect on the depression group and normal control group was tested *via* logistic regression (LR) and support vector machine (SVM) models, respectively, both of which were used frequently in previous studies. Due to its superior discriminant effect, the LR model was selected for further analysis. All machine learning processes were completed with scikit-learn in Python 3.8. All parameters were performed by default to avoid the influence of manual intervention on discrimination results. The f_classif method in the feature_selection module of scikit-learn was used to screen all of acoustic features. The LR analysis was performed again based on the 20 selected variables, while analyzing the contribution of each variable to the classification. We performed a 10-fold cross validation of the classification and divided the dataset into the training set and the test set at a 7:3 ratio. The test data was only used for the validation of the discriminant model and were not used in any training process. Figures were generated from Matplotlib, a popular data visualization package for Python (38).

## Results

### General Demographic Data of the Two Groups

We enrolled 71 patients with depression and 62 healthy controls in Beijing, China. There were no significant differences in age, gender, or education between the two groups (Table 1). The depression group was accompanied by obvious symptoms of anxiety. The group’s psychic anxiety score of HAMA was 12.30 ± 5.12, while the somatic anxiety score of HAMA was 9.16 ± 6.00. The subscale scores of HAMD were 6.35 ± 3.13 (anxiety/somatization score), 0.57 ± 0.69 (weight score), 1.97 ± 1.44 (cognition score), 4.49 ± 2.02 (retardation score), and 2.86 ± 1.86 (insomnia score).

**TABLE 1:** Demographic and clinical characteristics of all participants *N* = 133.

Characteristics	Depression group (*n* = 71)	Control group (*n* = 62)	Group comparison	
			
	Mean ± SD	Mean ± SD	*t*/χ^2^	*p*
Age (years)	34.90 ± 9.32	36.67 ± 8.56	1.15	0.25
Gender (Male,%)	33.80%	31.30%	0.1	0.85
Education (years)	16.25 ± 2.05	15.86 ± 1.83	−1.17	0.24
Age of onset (years)	27.59 ± 6.25	NA	NA	NA
Disease duration (years)	7.31 ± 6.02	NA	NA	NA
HAMA score	21.3 ± 9.93	NA	NA	NA
HAMD score	16.09 ± 6.24	NA	NA	NA
PHQ-9 score	13.13 ± 6.61	NA	NA	NA

*HAMA, Hamilton Anxiety Scale; HAMD, Hamilton Depression Scale; PHQ-9, Patient Health Questionnaire.*

### Multivariate Analysis of Variance of Acoustic Characteristics Between Depression and Normal Control Groups

A multivariate analysis of variance (ANOVA)—with gender, age, and education level as covariates—showed significant inter-group main effects of all of the acoustic characteristics (*p* < 0.0001), which survived FDR-correction. For η^2^ 0.01, 0.06, and 0.14 were considered small, moderate, and large effect sizes, respectively. ZCR, F0, MFCC1-5, MFCC7-10 showed a large effect size, and the remaining features showed a moderate effect size. Only MFCC2, MFCC3, MFCC8, and MFCC9 had statistically significant conditional main effects in the positive, neutral, and negative emotion tasks (η^2^ = 0.06, 0.09, 0.05, and 0.02, respectively), but none of the acoustic characteristics had a group × condition interaction effect (Table 2).

**TABLE 2:** Differences in acoustic characteristics between the depression and control groups under different emotional tasks *N* = 133.

	Depression group (*n* = 71) Mean (*SD*)			Control group (*n* = 62) Mean (*SD*)			Group effect		
			
	Positive	Neutral	Negative	Positive	Neutral	Negative	*F*	*p*	η ^2^
ZCR (10^–2^)	4.91 (1.78)	5.08 (1.76)	5.01 (1.82)	6.34 (0.82)	6.67 (0.80)	6.63 (0.82)	128.07	<0.0001*	0.25
HNR (10^–2^)	34.59 (8.97)	35.78 (9.03)	35.14 (9.10)	31.30 (3.46)	31.78 (3.36)	31.52 (3.88)	34.09	<0.0001*	0.08
F0	38.68 (38.55)	44.23 (41.60)	41.26 (39.33)	13.61 (11.84)	17.60 (12.77)	15.67 (14.99)	85.13	<0.0001*	0.18
MFCC1	−6.01(5.25)	−6.54(5.07)	−6.64(5.13)	−10.88(1.49)	−11.73(1.45)	−11.68(1.46)	181.35	<0.0001*	0.32
MFCC2	−0.84(4.20)	0.81 (3.99)	0.70 (3.94)	−6.23(1.84)	−4.23(1.61)	−4.46(1.63)	265.72	<0.0001*	0.41
MFCC3	−2.69(2.72)	−1.24(3.01)	−1.27(2.76)	−5.99(2.26)	−4.16(2.30)	−4.28(2.34)	158.39	<0.0001*	0.29
MFCC4	−3.56(4.07)	−4.47(4.30)	−3.77(4.06)	−7.33(2.24)	−8.06(2.23)	−7.49(2.24)	119.36	<0.0001*	0.23
MFCC5	−5.92(4.79)	−6.50(4.55)	−6.04(4.65)	−9.26(2.37)	−9.55(2.32)	−9.32(2.31)	68.58	<0.0001*	0.15
MFCC6	−7.13(5.64)	−6.74(5.46)	−6.78(5.37)	−9.55(2.31)	−9.00(2.01)	−9.19(2.14)	28.66	<0.0001*	0.07
MFCC7	−4.49(3.08)	−5.14(2.95)	−4.80(2.98)	−8.19(2.68)	−8.84(2.27)	−8.51(2.32)	171.70	<0.0001*	0.31
MFCC8	−3.36(3.60)	−4.93(3.73)	−4.47(3.64)	−6.36(2.16)	−8.08(2.09)	−7.55(2.01)	99.82	<0.0001*	0.20
MFCC9	−3.14(3.24)	−3.95(2.63)	−3.78(3.05)	−5.11(2.07)	−5.97(1.59)	−5.83(1.67)	67.17	<0.0001*	0.15
MFCC10	−4.62(4.14)	−3.90(3.78)	−4.50(4.15)	−8.14(2.93)	−7.42(2.26)	−8.08(2.68)	130.32	<0.0001*	0.25
MFCC11	−3.98(2.57)	−3.68(2.95)	−4.02(2.94)	−5.55(2.30)	−5.00(2.42)	−5.40(2.42)	32.37	<0.0001*	0.08
MFCC12	−2.07(1.86)	−2.18(1.78)	−2.40(1.94)	−3.41(2.01)	−3.39(1.89)	−3.75(2.02)	48.35	<0.0001*	0.11

*Indicates survived FDR-correction.

*ZCR, zero-crossing rate; HNR, harmonic-to-noise ratio; F0, fundamental frequency; MFCC, mel-frequency cepstral coefficient.*

### Correlation Between Depressive Symptom Scores and Acoustic Characteristics of the Depression Group

Since there were significant differences between the groups for each acoustic characteristic indicator, we performed a Spearman correlation analysis for all acoustic features and the PHQ-9, HAMA, and HAMD scores. The results showed that MFCC4 and MFCC7 had significant positive correlations with the PHQ-9 score, regardless of the positive, neutral, or negative emotion tasks. ZCR in the positive condition task showed a significant correlation with the HAMA total score (*r* = 0.31) and with the HAMA somatic anxiety subscale score (*r* = 0.34). MFCC9 in the neutral task showed a negative correlation with the anxiety/somatization subscale score of the HAMD (*r* = −0.34). The remaining acoustic characteristics did not show a significant correlation with depressive symptoms (Table 3).

**TABLE 3:** The correlation between depressive symptom severity and acoustic characteristics.

	PHQ-9 score	HAMA total score	HAMA somatic anxiety score	HAMD anxiety/somatization score
ZCR-positive	–0.13	0.31*	0.34*	0.18
MFCC4-positive	0.39*	–0.06	0.02	–0.05
MFCC4-neutral	0.42*	–0.02	0.01	0.02
MFCC4-negative	0.36*	–0.10	–0.09	–0.09
MFCC7-positive	0.43*	0.10	0.11	–0.11
MFCC7-neutral	0.41*	0.14	0.17	–0.14
MFCC7-negative	0.46*	0.09	0.12	–0.05
MFCC9-neutral	–0.02	–0.09	–0.07	−0.34*

*p < 0.05.

*PHQ-9, Patient Health Questionnaire; HAMA, Hamilton Anxiety Scale; HAMD, Hamilton Depression Scale; ZCR, zero-crossing rate; MFCC, mel-frequency cepstral coefficient.*

### Linear Regression Analysis Between Clinical Symptom Scores and Acoustic Features

In linear regression, the PHQ-9 score was a dependent variable, while age, gender, education, MFCC4, and MFCC7 were independent variables. To avoid high collinearity of the same features under different emotion tasks, we selected MFCC4-neutral and MFCC7-negative, which had the highest correlation coefficient with the PHQ-9 score. Stepwise method was used. The result showed statistically significant effects, *F* = 7.25, *R*^2^ = 0.20, *p* = 0.01 (Table 4).

**TABLE 4:** Linear regression analysis between PHQ-9 score and MFCC7-negative.

	β	SE	*t*	*p*	95% confidence interval of β	
					Lower bound	Upper bound
**PHQ-9 score**						
(constant)	17.00	1.79	9.49	< 0.001	13.34	20.65
MFCC7-negative	0.90	0.33	2.69	0.01	0.22	1.58
**HAMD anxiety/somatization score**						
(Constant)	4.33	1.11	3.92	< 0.001	2.09	6.58
MFCC9-neutral	–0.45	0.22	–2.04	0.049	–0.91	–0.001

Then, the HAMD anxiety/somatization score was used as a dependent variable, while MFCC9-neutral, age, gender, and education were independent variables. Similarly, stepwise method was used. Here too, linear regression showed statistically significant effects, *F* = 4.15, *R*^2^ = 0.11, *p* = 0.049 (Table 4).

### Discriminant Analysis of Acoustic Characteristics of the Depression Group

**FIGURE 1:**
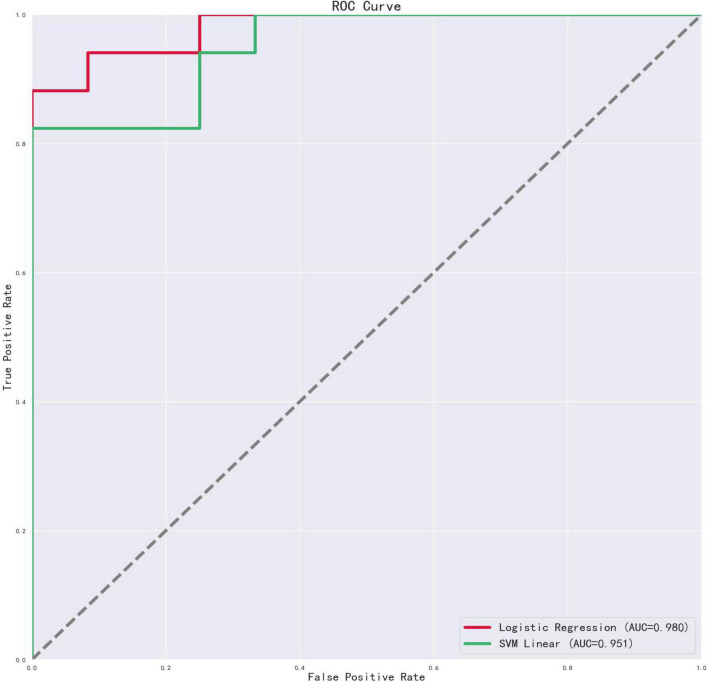
ROC curves of acoustic features in LR and SVM models. The red line represents LR model; the green line represents SVM model; the horizontal axis is the false positive rate, and the vertical axis is the true positive rate.

The LR model showed better discriminant results than SVM between the depression and control groups, with a discrimination accuracy of 89.66% and AUC of 0.98. A recall of 0.94 signified that 94% of depression patients were predicted as the depressed group, and precision of 0.89 signified that 89% of participants in the predicted depressed group were actually depression patients. Further, 83% of the healthy controls were predicted to be in the control group, and 91% of the participants in the predicted control group were actually healthy controls. The discrimination accuracy of the SVM model and the AUC were 86.21% and 0.95, respectively (see Table 5 and Figure 1).

**TABLE 5:** The discriminant result of grouping by different classifiers.

Indicator	LR		SVM	
	Depression group	Control group	Depression group	Control group
Precision	0.89	0.91	0.93	0.79
Recall	0.94	0.83	0.82	0.92
F1 score	0.91	0.87	0.87	0.85

*LR, Logistic Regression; SVM, support vector machine.*

**FIGURE 2:**
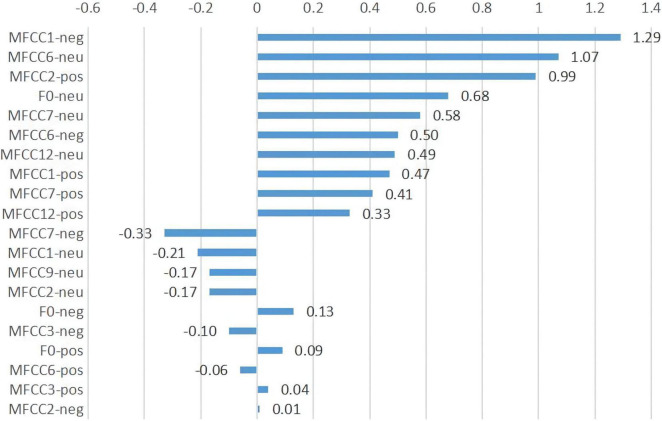
The 20 acoustic features with the largest contribution in LR model. pos, positive emotion task; neg, negative emotion task; neu, neutral emotion task.

Figure 2 shows the 20 acoustic features with the largest contribution to discrimination in the LR model. Concerning acoustic features—particularly MFCC1-negative, MFCC6-neutral—with contributions greater than 0, the larger the value, the more likely the participant is to be identified as depressed. In contrast, when these acoustic features have contributions less than 0, the smaller the value, the more likely the participant is to be identified as depressed.

## Discussion

There were significant differences in acoustic characteristics between the depression and normal control groups, after controlling for gender, age, and education. Only MFCC2, MFCC3, MFCC8, and MFCC9 showed differences during the positive, neutral, and negative emotion tasks. This suggests that there are differences in some acoustic features under different implicit emotional task patterns. Taguchi et al. also found that MFCC2 was higher in depressed patients than in normal controls, which is consistent with our results, but they did not report an association between MFCC and depressive symptom severity (5). These inconsistent results may be due to differences in language, disease stages, and speech recording methods. Another study from China found that MFCC5 and MFCC7 of the depression group were consistently lower than those of the control group across different emotional and situational tasks, and F0 was lower while ZCR was higher for the depression group in the reading text task, which was contrary to the findings of the present study (6). This inconsistency may be attributed to the different clinical characteristics of depression, such as anxiety and psychomotor retardation. The accuracy of identifying depression using acoustic characteristics was close to 90%. These results suggest that acoustic features may comprise a useful biomarker for diagnosing depression. Moreover, ZCR, MFCC4, MFCC7, and MFCC9 were associated with depressive and anxiety symptoms severity. MFCC7-negative can predict a PHQ-9 score; meanwhile, MFCC9-neutral can predict an anxiety/somatization score. Therefore, we believed that MFCC7 and MFCC9 can partly reflect the severity of depressive and anxiety symptoms and have the potential to be quantitative, objective indicators in evaluating disease severity.

Patients with depression often have autonomic nervous function disorders, and the muscle tone of their vocal tract can be affected by the disease, which may be one of the factors that distinguish depressed patients’ acoustic characteristics from those of healthy individuals. Patients with depression often feel tired, and a study suggests that sympathetic nervous system activity is enhanced, and parasympathetic nervous system activity is weakened during fatigue (28). Changes in the autonomic nervous system cause disturbances in the respiratory rate (29), and this may affect the airflow from the lungs to the vocal tract. Muscle tension is also a common symptom of depression (30). Changes in muscle tone can alter the dynamics of the vocal tract, limiting joint movement (31), which in turn slows speech and affects sound frequency. Air passes through the vocal cords and glottis, and the vocal tract acts as a filter that amplifies and attenuates different sound frequencies through movement, adjusting the spectrum of the glottis waveform (32). In a previous study, MFCC was associated with muscle movement of the vocal tract on MRI scans (16); MFCC can also reflect voice mood and is minimally affected by age and sex (5). We found that ZCR, MFCC4, MFCC7, and MFCC9 were associated with depression symptom severity. However, how depression affects muscle movement of the vocal tract that causes such changes in the specific frequencies of the patient’s voice remains unknown.

Speakers can constantly monitor and adjust their voice by using their speech as feedback in the auditory circuit (32). In the central auditory pathway, 5-hydroxytryptamine (5-HT) exists in many structures from the cochlear nucleus to the auditory cortex. It is one of the most important neurotransmitters in auditory processing; 5-HT neurons in the inferior colliculus can adjust the frequency of perceived tones by changing the response latency of neurons (33). Female patients with depression show reduced tolerance to sound, also known as auditory hypersensitivity (34). People with low expression of 5-HT transporter perceived sounds as clearer than those with high expression, showing a higher signal-to-noise ratio and volume (35). Therefore, patients with depression may also be more sensitive than healthy individuals when hearing the sounds of their own voices. Due to the low 5-HT function, the perceived frequency of their own sounds may change. Through auditory feedback, patients may tend to have lower intonation and acoustic features, which are different from those of normal controls when speaking.

Rats express their emotions through ultrasonic vocalizations, and different emotions correspond to different frequencies of ultrasound; ultrasonic vocalizations in anxious conditions due to isolation are associated with 5-HT. Drugs that act on the 5-HT receptor, such as some serotonin reuptake inhibitors and serotonin 5-HT_1A_ receptor agonists, dose-dependently reduce isolation-induced ultrasound vocalizations in rats (36). Serotonin reuptake transporter gene-deficient rats exhibited more anxious behavior, accompanied by changes in the ultrasonic vocal structure, and they were less likely to make orectic 50 kHz vocalizations when exploring novel environments (37). The above studies on animals suggest that 5-HT may be related to the frequency and behavior of the sound emitted. However, there are no studies on human vocalization and 5-HT. Given that 5-HT plays an important role in the pathogenesis of depression, our results lead us to suspect that 5-HT may be involved in the pathological mechanism of the abnormal acoustic characteristics of depressed patients.

### Limitations

Although the findings of this study have implications for improving the diagnosis of depression, there are some inherent limitations to the study design. First, the exploratory cross-sectional design of this study precluded causal inferences. Longitudinal follow-ups of patients with depression, including observation of the entire course of disease with and without drug treatment, need to be carried out in the future. Second, the clinical symptom assessment in this study was simple; future studies should include more dimensions of depression, including the assessment of suicide risk and autonomic nervous function. Third, the experimental paradigm of this study was relatively simple and warrants further expansion to simulate different scenarios, help gain a comprehensive understanding of the changes in the acoustic characteristics of depressed patients, and fully evaluate their accuracy as objective biomarkers of depression. Furthermore, this study only included Chinese Mandarin samples, whether the specific language or other cultural factors may influence the generalizability of the results is still unclear. Finally, although we performed a 10-fold cross validation, a larger sample size and independent validation datasets may be more effective for verifying our conclusions.

## Conclusion

The change in acoustic characteristics in patients with depression may not be caused by a single factor, and the specific underlying mechanism remains unclear. However, the acoustic characteristics of depressed patients were significantly different from those of the normal control group in this study, and the accuracy of depression classification based on acoustic features was very high. These findings suggest that acoustic characteristics may become objective biomarkers of depression; this may help clinicians make objective diagnosis of depression, reduce the rate of missed diagnosis and misdiagnosis, and promote the development of Internet medical treatment.

## Data Availability Statement

The raw data supporting the conclusions of this article will be made available by the authors, without undue reservation.

## Ethics Statement

The studies involving human participants were reviewed and approved by the Ethics Committee of Beijing Huilongguan Hospital. The patients/participants provided their written informed consent to participate in this study.

## Author Contributions

S-PT, Z-RW, and Y-LT contributed to the conception and design of the study. QZ performed the statistical analysis and wrote the first draft of the manuscript. S-PT reviewed and revised the manuscript. H-ZF, Y-LZ, and Z-XT organized the database. Y-LL, LL, and Y-XW contributed to provision of study materials and patients. All authors contributed to the manuscript revision, as well as read and approved the submitted version.

## Conflict of Interest

The authors declare that the research was conducted in the absence of any commercial or financial relationships that could be construed as a potential conflict of interest.

## Publisher’s Note

All claims expressed in this article are solely those of the authors and do not necessarily represent those of their affiliated organizations, or those of the publisher, the editors and the reviewers. Any product that may be evaluated in this article, or claim that may be made by its manufacturer, is not guaranteed or endorsed by the publisher.
